# Research protocol of a multifaceted prospective mixed-method longitudinal study: Navigating Through Life – Western Australian study of transitions from out-of-home care

**DOI:** 10.1186/s12889-020-09138-x

**Published:** 2020-07-29

**Authors:** Lauren Parsons, Donna Chung, Reinie Cordier, David Hodgson, Stephan Lund, Philip Mendes, Melissa O’Donnell, Richard Parsons, Stian Thoresen

**Affiliations:** 1 School of Occupational Therapy, Social Work and Speech Pathology, Faculty of Health Sciences, Curtin University, Bentley, Western Australia Australia; 2 Department of Social Work, Education and Community Wellbeing, Faculty of Health and Life Sciences, Northumbria University, Newcastle, UK; 3 Division of Social Work, School of Allied Health, Faculty of Health and Medical Sciences, University of Western Australia, Perth, Western Australia Australia; 4 Department of Social Work, Faculty of Medicine, Nursing and Health Sciences, Monash University, Caulfield, Victoria Australia; 5 Telethon Kids Institute, Perth, Western Australia Australia

## Abstract

**Background:**

Developing robust evidence is a challenge for researchers working with disadvantaged or vulnerable populations. For example, research shows that young people who have transitioned from out-of-home care (OOHC) to independent adulthood often experience poor long-term outcomes. However, evidence for the aetiology of those outcomes is weak due to methodological limitations such as small sample sizes and a lack of longitudinal data. This paper details the protocol for Navigating Through Life, a study that utilises novel research methods to better understand the pathways and outcomes of young people as they leave OOHC in Western Australia (WA)*.*

**Methods:**

Navigating Through Life is a longitudinal, mixed methods, population-based study. A prospective longitudinal study of young people aged 15–25 years will follow participants’ experiences and outcomes over a two-year period. Quantitative and qualitative data is being collected from participants five times over 2 years, using standardised outcome measures and individual interviews. Outcome measures focus on key dimensions of young people’s lives (e.g., social inclusion, well-being, resilience, self-determination). Interviews examine important influences and the variable contexts into which young people have transitioned from care. In addition, retrospective population-level data for young people transitioning from OOHC will be obtained from linked Western Australian government administrative records. Using a multitude of data sources, analysis will map pathways and outcomes of young people with care experience, and comparisons will be made with other population groups within WA.

**Discussion:**

Navigating Through Life exemplifies a novel utilisation of multiple data sources to research outcomes for vulnerable and difficult to reach populations, and offers insights for other complex mixed-methods longitudinal studies. Results will provide new and more comprehensive data about specific pathways that may be influential to a range of post-care outcomes. Findings will extend evidence to inform better service-delivery models that improve outcomes and reduce disparities for vulnerable young people.

## Background

Out-of-home-care (OOHC) is accommodation, care and support provided for children aged 0–17 years who are unable to live with their parents [1]. Children may be removed from their parents when they have experienced physical, sexual, or emotional abuse or neglect, are at substantial risk of harm, or when their parents are unable to provide care for any other reason (e.g. medical conditions). OOHC is authorised and funded by the state and may include foster care, residential care, family group homes, or kinship care arrangements [1]. Leaving care, which is our concern, marks the cessation of legal responsibility by the state for young people living in OOHC, which generally occurs within Australia at no later than 18 years of age. Leaving care is a major life transformation, and a process that involves transitioning from dependence on state care accommodation and supports to so-called independence and self-reliance [2].

There is overwhelming evidence for the myriad vulnerabilities and disadvantages facing care leavers, and developing evidence that can improve outcomes for young people in and exiting OOHC is critical. Care leavers have diverse backgrounds and experiences of care and care leaving [3], but many experience disproportionately poor educational outcomes, homelessness, elevated contact with the justice or youth justice systems, and estrangement from culture and community [2, 4–6]. However, a lack of reliable and comprehensive data on the characteristics, experiences, and transition pathways of young people leaving care creates problems for adequately informing policy. For example, no precise Australian figures are available as to the proportion of care leavers exiting care from each category of care (i.e., foster/kinship care, residential care), or their particular transitional experiences and needs [1]. In addition, most Australian studies are based on State or Territory data using small samples, limiting the generalisability of the findings. Studies that include larger survey samples are limited by their focus on a single point in time, which does not reveal leaving care pathways and transitions over time in sufficient depth. This lack of longitudinal studies results in a dearth of indicators for how transitions may relate to other policy areas such as health, housing, and employment, or the needs of particular groups such as Aboriginal and Torres Strait Islander young people, or young people with disabilities [3]. The *Beyond 18* study is a recent exception, as it utilised a longitudinal survey, interview and data linkage methodology to examine post OOHC transitions, pathways and outcomes. However, their sample sizes diminished greatly over time, limiting the representativeness of the sample and therefore the generalisability of results [7]. Hence, there is a vital need for the development of more robust evidence that would document the varied and changing pathways of care leavers to inform the range of supports that are most required for meeting the varied needs of care leavers.

This paper describes the research protocol and methodology for the Navigating Through Life study; a large population-based prospective longitudinal study that will gather comprehensive quantitative and qualitative data on young people currently in and transitioning from OOHC in one Australian state, Western Australia. Navigating Through Life’s methodology is novel and comprehensive, combining retrospective administrative data with prospective quantitative and qualitative longitudinal outcome measurement. The protocol discussed forthwith marks an important step towards addressing the limitations of existing research into young people’s transitions from OOHC. Importantly, Navigating Through Life provides a template for researching with other vulnerable populations who, without improved interventions, are likely to continue to have poor outcomes into adulthood. The results of this particular study will be used for developing more targeted, evidence-informed leaving care programming. Replication of these methods could produce a similar evidence-base for new or improved service planning and provision for other marginalised and difficult to reach populations.

### Research aims

The research protocol described in this paper will generate a detailed body of findings, to provide quality evidence about young people’s experiences, overall wellbeing, and post care pathways. Navigating Through Life is addressing four interrelated aims:

1. To map longitudinal pathways and the lived experiences of young people as they transition to leave OOHC, and after exiting OOHC;
2. To identify key factors for meeting the cultural, social and developmental needs associated with successful transitioning from OOHC for young people;
3. To gain a population based understanding of how varied pathways are associated with particular outcomes of young people in and transitioning from OOHC; and
4. To identify Aboriginal family and community perspectives on the barriers and enablers important to the achievement of developmental milestones from a cultural perspective.

## Methods/design

To address the current limitations in research on transitions from OOHC, Navigating Through Life is bringing together both qualitative and quantitative data from an array of sources and across multiple time points in the lives of participants.

### Study setting

The study will be conducted in the state of Western Australia (WA). OOHC policy within WA is enacted by the WA Department of Communities, a State administered human services agency, via their Child Protection and Family Support services. Figures as of 30 June 2019, indicate there are 5379 children in OOHC in WA [8]. A majority of WA children in care (87%) are placed in home-based care (e.g., foster or kinship care), and around 7 % are placed in residential care [8]. Australian Aboriginal children are over represented within the care system nationally (163.8 per 1000 Aboriginal children); a rate of eight times that of non-Aboriginal children (19.7 per 1000), and 56% of children in care in WA are of Aboriginal descent [1, 8]. During 2018–2019, 256 young people aged 15 or older left care in WA. A majority of those young people who left care (70%) resided in the Perth metropolitan area, and the remaining 30 % were in regional and remote towns across the State [8].

The geography and population distribution of WA presents a unique challenge for service provision and research. Just over 10 % (2,595,192) of the Australian population (24,992,860) resides in WA, despite covering about one-third of the Australian landmass (253 million of 769 million hectares; 9). Just shy of 80 % of the WA population (2,059,484) live in the greater Perth area, the State Capital. The remaining 535,708 of WA’s residents populate an area exceeding 252 million hectares, a concentration of 0.002 residents per hectare [9]. Consequently, WA is a large and sparsely populated landmass, with large distances between cities, regional centres, remote, and very remote communities. This vast geography limits the possibility and availability of many services in remote and very remote areas, and presents a challenge for collecting research evidence that represents the experiences of young people across the state.

### Study design

Navigating Through Life is funded for 4 years, commencing April 2018. The project is comprised of three interrelated studies that address the research aims. A prospective mixed-method longitudinal study (Longitudinal Study) will be conducted with a subset of young people aged 15–25 years who have lived experience of OOHC in WA (Aims 1 and 2). Quantitative and qualitative data will be collected from two distinct cohorts: 1) an *In-care Cohort* of young people aged 15–17 years who are in care when they enter the study, and 2) an *Exited-care Cohort* of young people aged 18–25 years who have exited care when they enter the study. Young people will participate in face-to-face, phone or video-conference interviews at five time points over a two-year period.

**Fig. 1:**
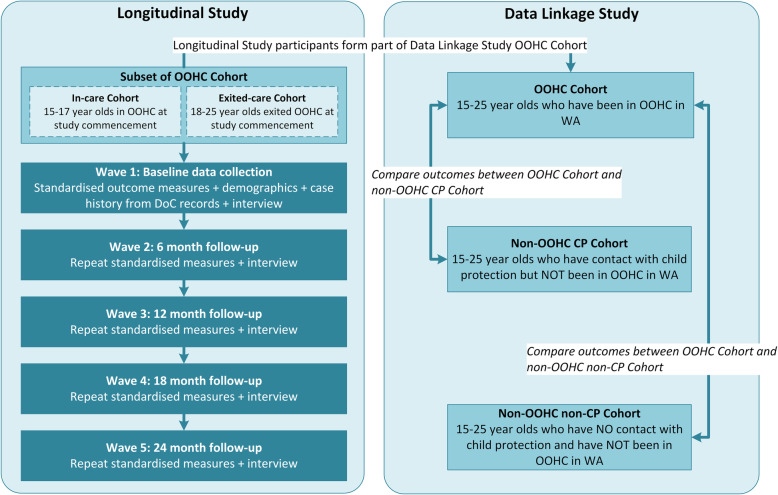
Study design

A concurrent Data Linkage Study will utilise population-level data obtained from linked WA government administrative records (Aims 1 and 3). WA has a long history of data linkage with health and mental health data linked for the WA population from 1970 onwards through the WA Data Linkage System [10]. For the purpose of this project, administrative records of all young people in WA will be obtained, and three distinct population groups will be examined: 1) all young people in WA with a care experience (OOHC Cohort); 2) all young people in WA who have interacted with the child protection system but were not placed in care (non-OOHC CP Cohort), and 3) young people in WA who have no interactions with the child protection system (non-OOHC non-CP Cohort). Prospective quantitative and qualitative data from the Longitudinal Study will be combined with retrospective linked data to provide comprehensive data about specific pathways that are influential to a range of post-care outcomes. Figure 1 depicts the nexus between the two studies.

A third study will investigate the perspectives of Aboriginal young people in OOHC, those who have left OOHC, their families and community in a regional or remote part of WA (Aims 2 and 4). This study will complement the population-level studies to provide an up-close and in-depth examination of Aboriginal young people’s experiences in their community context. The research protocol for this study is under development via co-design with Aboriginal people and researchers in the WA community. As such, the methodology of that study will not be described within this paper.

### Stakeholder involvement

Two Expert Reference Groups and a stakeholder Project Advisory Group (PAG) oversee Navigating Through Life and will provide guidance across its lifetime. A Lived Experience Expert Reference Group (LEERG) comprised of up to 10 young people who have transitioned from OOHC give a voice to young people, ensuring their experiences and knowledge are informing research practices and knowledge translation strategies. This approach with young people is well established in research within Australia [11]. An Aboriginal Expert Reference Group (AERG) comprised of up to 10 representatives from Aboriginal Controlled Community Organisations provides lived and Aboriginal experience, perspectives, knowledge and direction to the research. The AERG ensures culturally sensitive research practices are implemented, and are involved in the co-design of the Aboriginal Place-Based Study. The PAG is comprised of representatives from key government and non-government organisations within the OOHC and youth sectors in WA (e.g., advocacy, service provision, policy development and research), along with two members from the LEERG and AERG. The role of the PAG is to provide strategic leadership in the implementation and sustainability of the research project, including the engagement and retention of young people as study participants. All groups meet every 3 months, or more frequently if required.

### Participants

#### Longitudinal study

##### Inclusion and exclusion criteria

To participate in the Longitudinal Study young people must meet two inclusion criteria: 1) have spent at least 6 months in OOHC, and 2) be aged 15–25 years at the time of entering the study. Young people will be excluded from the study if they have a significant disability that prevents them from providing informed consent or participating in the data collection activities.

##### Sample size

The target sample size for the Longitudinal Study is *n* = 338. Power calculations determined the required sample for a large effect size (*d* = 0.80) is *n* = 138. Calculations were based on a repeated measures ANOVA with four groups (OOHC Cohort, non-OOHC CP Cohort, non-OOHC non-CP Cohort, Longitudinal Study Participants), *α* = 0.05, power = 0.9, and correlation between repeated measures = 0.5. However we will oversample by 245% at Wave 1 to allow for a 20% attrition rate at each subsequent wave. To ensure similar numbers of participants who are in OOHC (In-care Cohort) and who have transitioned out of care (Exited-Care Cohort) at Wave 1, we aim to recruit 169 young people for each cohort. This number is also large enough to allow for within-group regression analysis. We will monitor the socio-demographic characteristics of the sample during recruitment with the aim of obtaining a representative sample according to age, gender, geographical location (i.e., metropolitan, regional, remote), and cultural identity (i.e., Aboriginal and non-Aboriginal young people).

##### Recruitment

Recruitment of Longitudinal Study participants commenced in April 2019, and approximately 20% of the intended sample has been recruited to date. A multi-faceted approach to recruitment is underway with the assistance of multiple government and non-government agencies across the WA OOHC and youth service networks and advocacy bodies. A range of recruitment strategies are being deployed, dependent on whether potential participants are in the In-care or Exited-care Cohorts, and the supports young people are receiving at the time of recruitment.

The Child Protection and Family Support agency within the WA Department of Communities is assisting with recruitment. Child Protection workers inform young people on their caseloads about the study and refer them to researchers with the young person’s agreement. The research team are visiting Child Protection and Family Support offices to inform Child Protection workers about the study and to negotiate suitable strategies to disseminate information to young people and put young people in contact with researchers. Strategies to date include information sessions with young people, mail-outs, and the designation of a ‘Project Champion’ within each office to promote the study to colleagues and act as a point of contact for the research team and potential participants.

There are three specialist leaving care service providers in WA who provide transitional support to young people who have been in OOHC until they reach 25 years of age. Similar to the approach within the WA Department of Communities, researchers are presenting information about the research to agency staff so that frontline practitioners can invite young people to participate in the study. In addition, researchers have partnered with relevant OOHC agencies and providers to run ‘drop-in sessions’ where potential participants have an opportunity to meet the researchers, learn about the study, and participate in the first wave of data collection.

Researchers are contacting other stakeholder groups within the OOHC sector to assist with recruitment. Key groups include the CREATE Foundation (an advocacy body for children and young people with care experience), the Foster Care Association of WA (peak body for foster families), and the Youth Advisory Council of Western Australia (advocacy body for young people and the non-government youth sector). The research team ask for recruitment information to be disseminated through their member networks via newsletters, social media, and websites.

Generic youth service providers, including housing and homelessness services, will be contacted by peer-researchers (young people with lived experience of care or homelessness) and requested to assist with recruitment. Peer researchers will be trained and supported by the research team in ways to engage service providers in recruitment for the study. Service providers will be asked to review the service users they support to identify anyone who may fit the study criteria and refer them to the research team. Recruitment support may also include displaying recruitment advertisement materials, a member of the research team being ‘at hand’ at a drop-in centre if a service user who meets the study inclusion criteria would like more information about the study or is willing to participate.

Finally, broad recruitment strategies have been deployed, including a Navigating Through Life website and Facebook page, snowballing by asking study participants to refer others they may know who meet the eligibility criteria to the research team, and through recruitment flyers to students at the local universities and technical colleges.

##### Retention

Research participant retention is a major challenge for longitudinal studies, especially studies that include vulnerable populations such as young people in and leaving OOHC. Retention rates can be difficult to predict, however the *Longitudinal Surveys of Australian Youth* (LSAY) and *Beyond 18* study provide a useful benchmark for predicting likely retention rates within Navigating Through Life. The LSAY include cohorts of nationally representative samples of Australian young people recruited at the age of around 15, and has retained 40–64% of their participants at wave five, with the most marked attrition occurring between the first and second wave [12]. The *Beyond 18* study, a longitudinal study of Australian care leavers in the state of Victoria, has retained 43% of participants at wave two, reducing to 30% at wave three [7, 13, 14].

Given that care leavers are likely a more difficult participant group to retain over time compared with other young Australians, a number of strategies will be implemented within Navigating Through Life to increase the likelihood of participant retention. First, waves will be conducted at 6 month intervals (compared to annually for LSAY and Beyond 18), as care leavers can be highly transient and researchers may lose touch with participants over a longer period of time. Data collection will be carried out in-person (rather than via postal survey), so that participants can develop a sense of trust with researchers. Lastly, participants will receive recompense for their time in the form of gift vouchers, as this form of recognition has been attributed to high retention rates in other longitudinal studies [15]. Vouchers will be valued at $40 at the first wave and increase in value by $20 for each subsequent wave in recognition of the increasing effort required of participants to remain connected to the research.

#### Data linkage study

##### Sampling

The Data Linkage Study sample will comprise all young people born in WA between 1993 and 2008 (i.e., aged 15–25 years at the start of the study), with their data provided until 2019. The sample will be identified using the Birth Registrations and Midwives Notification System. Three distinct cohorts within the sample will be identified for comparison: 1) young people with at least 6 months experience of OOHC (OOHC Cohort); 2) young people who have interacted with the child protection system but not placed in care (non-OOHC CP Cohort), and 3) young people who have no interactions with the child protection system (non-OOHC non-CP Cohort). The WA Department of Communities (Child Protection and Family Support) data will be used to identify these cohorts via the presence or absence of child protection involvement and OOHC across a young person’s life-course.

### Data collection procedures

#### Longitudinal study

The Longitudinal Study has two main data sources. Participants complete a series of self-report standardised outcome measures and qualitative interviews across five waves of data collection over a two-year period. The WA Department of Communities will provide researchers with administrative data pertaining to consenting participants. Administrative data will relate to participants’ interactions with the WA Department of Communities and tools used by Case Managers to identify the needs of young people and plan and implement support provided by the Department. The data collection schedule, constructs measured, data sources, and data collection instruments are detailed in Table 1.

**Table 1:** Measurement tools, data sources and constructs to be measured during the Longitudinal Study

Construct areas	Measurement tools	Source	Wave 1	Wave 2	Wave 3	Wave 4	Wave 5
Resilience	CD-RISC [16]	P	✓	✓	✓	✓	✓
Self-determination	AIR Self Determination Scale [17]	P	✓	✓	✓	✓	✓
Wellbeing	Strong Souls [18]	P	✓	✓	✓	✓	✓
Adverse experiences	ACEs Questionnaire [19]	P, DoC	✓	✓^a^	✓^a^	✓^a^	✓^a^
Independent living (IL) skills	IL Questionnaire	P	✓	✓	✓	✓	✓
Social Inclusion	Social Inclusion Questionnaire	P	✓	✓	✓	✓	✓
Living arrangements	Qualitative interview	P	✓	✓	✓	✓	✓
Leaving care planning	Qualitative interview	P	✓	✓	✓	✓	✓
Social and family relationships	Qualitative interview	P	✓	✓	✓	✓	✓
Education and training	Qualitative interview	P	✓	✓	✓	✓	✓
Employment	Qualitative interview	P	✓	✓	✓	✓	✓
Living costs	Qualitative interview	P	✓	✓	✓	✓	✓
Service use	Qualitative interview	P	✓	✓	✓	✓	✓
Background and culture	Qualitative interview	P	✓	✓	✓	✓	✓
Parenting	Qualitative interview	P	✓	✓	✓	✓	✓
Demographics	DoC administrative data	DoC	✓				
Period(s) of care	DoC administrative data	DoC	✓				
Investigations and outcomes	DoC administrative data	DoC	✓				
Safety	Care Plan, NAT, Viewpoint	DoC, CM, P	✓		✓		✓
Care arrangements	Care Plan, NAT, Viewpoint	DoC, CM, P	✓		✓		✓
Health	Care Plan, NAT, Viewpoint	DoC, CM, P	✓		✓		✓
Education	Care Plan, NAT, Viewpoint	DoC, CM, P	✓		✓		✓
Social and family relationships	Care Plan, NAT, Viewpoint	DoC, CM, P	✓		✓		✓
Recreation and Leisure	Care Plan, NAT, Viewpoint	DoC, CM, P	✓		✓		✓
Emotional and Behavioural development	Care Plan, NAT, Viewpoint	DoC, CM, P	✓		✓		✓
Identity and Culture	Care Plan, NAT, Viewpoint	DoC, CM, P	✓		✓		✓
Legal and financial matters	Care Plan	DoC, CM, P	✓				

*Notes. CD-RISC* Connor-Davidson Resilience Scale, *ACEs* Adverse Childhood Events, *P* Participant self-report, *DoC* Department of Communities administrative records, *NAT* Needs Assessment Tool, *CM* Case Manager report measure; ^a^ Participants will only complete if they are aged ≤ 18 years

##### Outcome measures and interviews with participants

The chief investigators (CIs), a post-doctoral researcher, a PhD student, and post-graduate research assistants are collecting Longitudinal Study data. In addition, peer-researchers with lived experience of OOHC have been trained by CIs in data collection methods and interview techniques. Researchers meet with young people face to face - including travelling to regional and remote locations in the WA - to administer outcome measures and conduct interviews. Outcome measures are administered via iPads using REDCap, a web based platform for instrument administration and data management. Digital audio recordings of interviews are collected and transcribed verbatim for analysis. Support for literacy and comprehension is provided to young people as needed, and tailored to individual needs.

*Outcome Measures*: The measures selected for use in Navigating Through Life cover six key dimensions of young people’s lives: 1) independent living skills; 2) social inclusion; 3) resilience; 4) self-determination; 5) emotional wellbeing, and 6) adverse childhood events. A summary of the longitudinal outcome measures are detailed in Table 2. Measures were selected if they were developed for use with young people, and have validated psychometric properties.

**Table 2:** Description of Longitudinal Study outcome measures

Measure Name ***Dimension Measured***	Items; Response Scale (Sub)scales	Psychometrics Validation Population	Administration schedule
Connor-Davidson Resilience Scale (CD-RISC) [16] *Resilience*	25 items; 5-point scale Overall scale	Internal consistency: α = 0.89 [16] Adults, young people; general population, trauma, mental health in- and out-patients.	Waves 1–5:all participants
American Institutes for Research (AIR) Self-Determination Scale [17] *Self-determination*	12 items; 5-point scale Capacity, Opportunity Overall scale	Internal consistency: split-half correlations = 0.95 [17] Test-retest reliability over 3-months: *r* = 0.74 [17] Children and young people aged 6–25 years, from a range of ethnic and socioeconomic backgrounds	Waves 1–5:all participants
Strong Souls [18] *Social and emotional wellbeing*	25 items; 4-point scale Anxiety, Depression, Suicide Risk, Resilience, Overall scale	Internal consistency: α = 0.70 [18] Structural validity: 4 factor structure [18] Australian Aboriginal young people	Waves 1–5:all participants
Adverse Childhood Experiences (ACEs) Questionnaire [19] *Abuse, neglect or household challenges*	10 items^a^; 3-point scale^b^ Overall scale	Internal consistency: α = 0.88 [20] Young people and adults, from a range of ethnic, socioeconomic and trauma backgrounds	Wave 1: all participants Wave 2–5: participants aged ≤18 years.
Independent Living Skills Questionnaire *Independent living skills*	42 items; 5-point scale Overall scale	To be determined though this project Young people with OOHC experience	Waves 1–5:all participants
Social Inclusion Questionnaire *Social inclusion*	37 items; 5-point scale Overall scale	To be determined though this project Young people with OOHC experience	Waves 1–5:all participants

^a^Items relating to experiences that are known to have occurred within a participant’s childhood (based on administrative data) will be removed prior to administration. This practice aims to reduce the risk of participant distress while completing the questionnaire;

^b^Response scale adapted for the purpose of Navigating Through Life. Original response scale = yes/no; additional option added where participants can indicate they have knowledge of an experience (e.g., were told about it by another person) but do not personally recall the event occurring

We were unable to find a validated measure of independent living skills, or a measure of social inclusion that was validated and developed for use with young people. As a result, we developed a measure of independent living skills and a measure of social inclusion that will be validated during the process of this research. To develop a measure of independent living skills, items from the Ansell-Casey Life Skills Assessment [21] and a checklist developed for use by the New South Wales Government Department of Communities and Justice [22] were collated and reviewed by three researchers to shortlist for inclusion in the new measure. The same process was undertaken to develop the measure of social inclusion using items from the SCOPE Long Form [23], Social Connectedness Scale [24], and Social Capital and Cohesion Scale [25]. Items were included in each measure if they represented a unique aspect of the construct to be measured, with minimal complexity to the language within the item. Wording of shortlisted items for both measures was reviewed and adapted to ensure all items were presented as statements, and a response scale was developed whereby participants indicate the strength with which they agree with each statement (i.e., 1 = strongly disagree - 5 = strongly agree). The draft measures was reviewed by the AERG and AG and members provided feedback on item relevance (i.e., were all items relevant to the concept being measured?), comprehensiveness (i.e., are any key elements relevant to the concept missing?) and comprehensibility (i.e., are items likely to be understood by young people?). Items were then adapted based on AERG and AG feedback and piloted with six young people. Feedback on item relevance, comprehensiveness and comprehensibility was collected from young people and items for both measures were adapted based on feedback to create the final versions for use in the study. The psychometric properties of both measures will be evaluated during the course of the study.

*Qualitative interviews:* Researchers conduct qualitative interviews with participants following completion of the standardised outcome measures. Interviews are audio-recorded and transcribed verbatim for analysis. Initially the interview schedule covers a broad range of life domains: current living arrangements, planning for independent living (for participants aged ≥17 years), connections to friends and family, school and post-school education or training, living costs, health and other services, family background and cultural identity, and parenting (for participants who are parents). Themes emerging from Wave 1 interviews will inform the development of the Wave 2 schedule and so on, so that themes can be developed and explored longitudinally across the study.

##### WA Department of Communities data

For consenting participants, administrative data will be collected from the Department of Communities database. Data concerning participants’ interactions with the WA child protection system, their most recent Care Plan, and responses to two assessment tools administered to Case Managers and young people are being provided by the Department of Communities.

*Child Protection Data:* Administrative data pertaining to participant’s child protection service interactions has been extracted from the Child Protection and Family Support database and provided to researchers. Extracted data includes participant demographics, the beginning and end dates of each care period, reasons for entering and exiting care, living arrangements associated with their care periods, harm or maltreatment investigations, reasons for and outcomes from those investigations, and any interactions participants may have had with child protection services since leaving care.

*Care Plans, Needs Assessment Tool (NAT), Viewpoint:* Care Plans, the NAT and Viewpoint are administrative tools completed during the course of a young person’s time in care. Care Plans detail ways in which the young person will be supported by the Department of Communities to meet their individual needs. The NAT is a survey of the Case Manager’s views about a young person’s behaviour and subsequent support requirements. Viewpoint is a self-report tool administered by the Department of Communities to capture a young person’s thoughts and feelings about aspects of their care experience. Items within all three tools are arranged under eight domains of child wellbeing, with Care Plans having one additional domain related to legal and financial matters. Table 3 describes how these eight domains are operationalised within each of the three tools.

**Table 3:** Operationalisation of domains of child wellbeing within Care Plans, the NAT, and Viewpoint

Domain of child wellbeing	Care Plans	NAT	Viewpoint
Safety	Qualitative review of previous Care Plan aspects relating to this domain, description of current safety needs or concerns, decisions about future actions to meet safety needs.	Young person’s actions or behaviours that place themselves or others at risk or harm.	Young person’s sense of physical and emotional security, and perceptions of own risk taking behaviour.
Care Arrangements	Qualitative review of previous Care Plan aspects relating to this domain, description of current care arrangement needs or concerns, decisions about future actions to meet care arrangement needs.	Amount of additional regular car travel required to support young person’s needs.	Young person’s satisfaction with current care arrangement and their participation in care planning, including leaving care planning if relevant.
Health	Qualitative review of previous Care Plan aspects relating to this domain, description of current physical and/or emotional health needs or concerns, decisions about future actions to meet health needs.	Level of impact of an identified mental, physical or developmental need on young person’s daily functioning.	Young person’s perception of own physical health, frequency of mental health symptoms.
Education	Qualitative review of previous Care Plan aspects relating to this domain, description of current education needs or concerns, future actions to meet health needs.	Young person’s level of educational attainment and attendance, and impact of any problem behaviours on schooling.	Young person’s school attendance rate, perception of school and academic attainment, perception of support provided (e.g., help with school work, acquiring materials and equipment).
Social and Family Relationships	Qualitative review of previous Care Plan aspects relating to this domain, description of current family and social relationship needs or concerns, decisions about future actions to meet needs around supporting and maintaining desired relationships.	Presence or absence of young person’s contact with immediate family members (i.e., parents and siblings).	Young person’s sense of closeness to carers and family members, satisfaction with family contact, presence of close friendships, satisfaction with contact with friends.
Recreation and Leisure	Qualitative review of previous Care Plan aspects relating to this domain, description of current recreational needs or desires, decisions about future actions to meet health needs.	Level of support required for young person to pursue recreational interests of choice.	Young person’s satisfaction with current levels of participation in and recreation and leisure activities.
Emotional and Behavioural Development	Qualitative review of previous Care Plan aspects relating to this domain, description of current emotional and behavioural concerns or needs, decisions about future actions to meet developmental needs.	Level of impact of an identified emotional need on young person’s daily functioning.	Young person’s perception of self, and self esteem.
Identity and Culture	Qualitative review of previous Care Plan aspects relating to this domain, description of current needs or desires with regards to connecting to cultural background, decisions about future actions to meet cultural needs.	Level of support required for young person to remain connected to cultural community	Young person’s perception of own depth of knowledge about family background and culture, satisfaction with assistance to learn family’s language, follow own religion, beliefs or culture
Legal and Financial	Qualitative review of previous Care Plan aspects relating to this domain, description of current legal and financial status, decisions about future actions to meet legal and financial needs.	NA	NA

Note. *NAT* Needs Assessment Tool, *NA* Not Applicable

Care plans are revised annually in conjunction with the young person, their case manager, and any other significant individuals involved in the care of the young person (e.g., carer, education officers, psychologist). The Department of Communities provides researchers with participants’ most recent care plans, and a data extraction form has been developed to categorise the information contained within each plan. Data relating to the identified needs, service use and goals of the young person, and planning decisions made to meet these needs in the future will be extracted according to the nine domains of child wellbeing within the plan. Data will be extracted into the forms by one researcher, and a second researcher will also extract a random overlap of 40% to determine interrater reliability.

The NAT contains 26 items, each with a unique set of categorical responses. Case Managers complete the NAT when a child or young person is first placed in care, and then again on an annual basis. The Department of Communities is providing researchers with the most recent NAT item responses for Longitudinal Study participants.

Viewpoint has been administered in WA since 2011. Items within the Viewpoint survey are presented as statements and young people provide responses on a 4-point scale to indicate level of agreement with the statement. Young people are provided an opportunity to complete Viewpoint on an annual basis. An individual’s responses are used to assist with individual care planning, and all young people’s responses are grouped and reviewed to inform service delivery decisions. The Department of Communities is providing researchers with Viewpoint data for Longitudinal Study participants at three time points; wave 1 (all past Viewpoint responses), 3 (any new responses since wave 1) and 5 (all new responses since wave 3).

#### Data linkage study

**Fig. 2:**
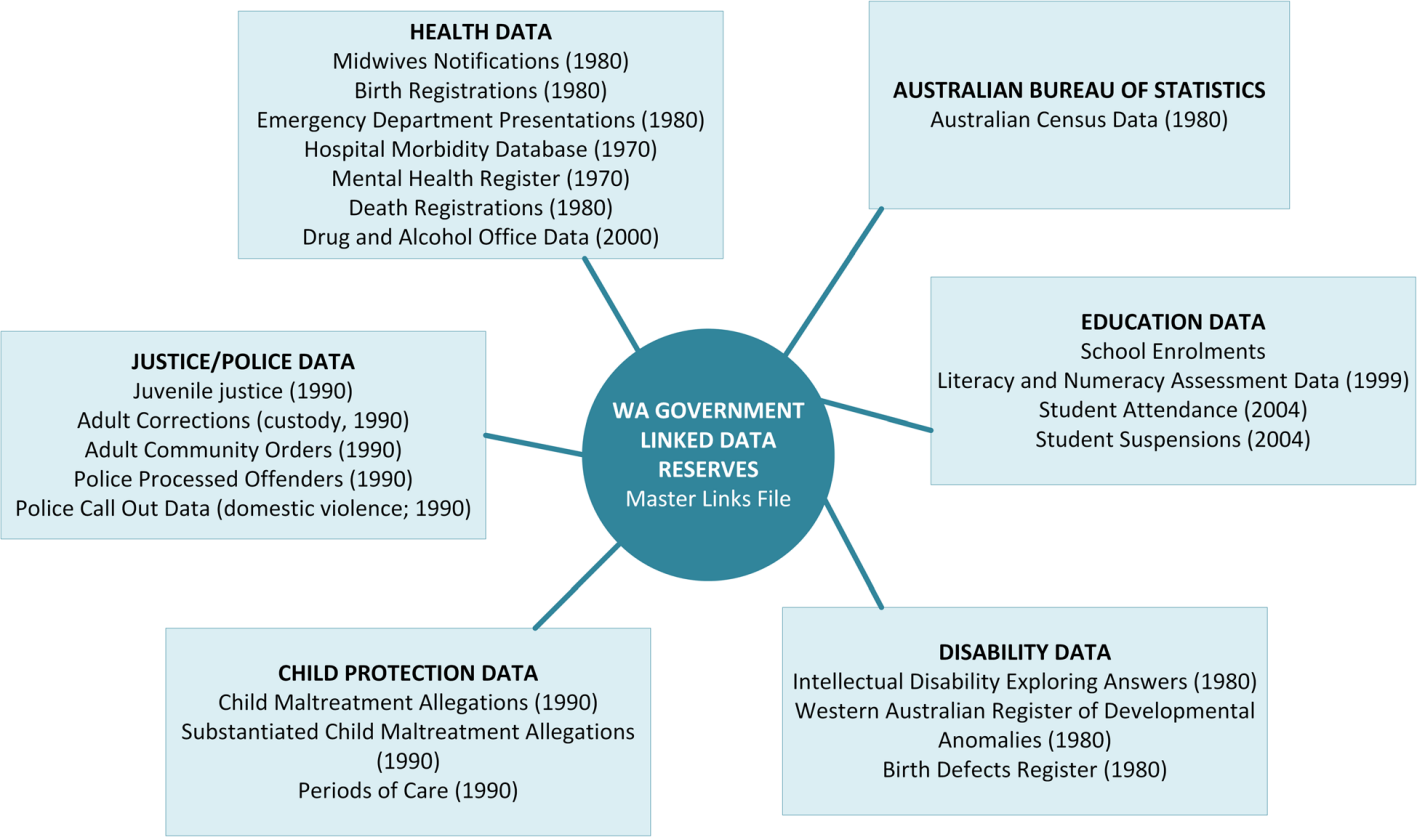
Linked databases within the WA Data Linkage System

Data for the Data Linkage Study will be sourced from the linked administrative data reserves created and maintained by WA Government. There are currently two reserves of de-identified linked data: 1) the WA Data Linkage System (maintained by the WA Department of Health; comprising predominantly health datasets) and, 2) the Social Investment Data Resource (maintained by WA Treasury; comprising predominantly non-health datasets). Applications to WA Government are underway to access datasets across departments, including Health, Child Protection and Family Support, Disability, Education, Justice, and Police to enable the examination of trajectories across multiple government service agencies. Figure 2 details the databases of linked data established in WA. The WA Data Linkage System also has additional capabilities: 1) the unique ability to provide genealogical links, enabling the investigation of parental factors that may impact on the pathways of their children; and 2) geocoding of addresses, enabling linkage to geographically-based socioeconomic indices from Australian census data.

Only de-identified data will be supplied to the research team. The Birth Registrations and Midwives Notification System will provide information on participant’s birth outcomes, maternal and paternal characteristics, and pregnancy complications. The WA Department of Communities (Child Protection and Family Support) data will identify child protection involvement across the participant’s life-course including child concern reports, maltreatment allegations, substantiated allegations and periods of OOHC. Outcomes data related to mental health and wellbeing, educational development, social development, employment and independent living will be sourced across the other available administrative datasets. The data will also enable the identification of geographic variation, pre-care and care-related factors, in order to understand the aetiology of outcomes for OOHC leavers with comparison to their birth cohort (see Table 4). Consent will be obtained from Longitudinal Study participants to enable identification of their linked administrative data via an encrypted identifier.

**Table 4:** Outcomes being investigated within the Data Linkage Study, databases utilised and how the outcomes are operationalised

Outcome Areas	Databases	Operationalised
Justice	Police Processed Offenders Police Call Out Data Juvenile Justice Adult Corrections	Age of offences, sequence of offending and pathways through justice system. Types of offences and orders, diversionary processes, incarceration.
Education	School Enrolments Literacy and Numeracy Assessment Data Student Attendance Student Suspensions	Literacy and numeracy achievement, high school completion, school attendance and suspensions.
Health	Hospital Morbidity Database Emergency Department Data Death Register	Timing and frequency of hospital admissions and emergency department presentations, length of stay, major health diagnoses, external cause of injuries.
Substance use	Mental Health Register Hospital Morbidity Alcohol and Drug Services Justice and Police Data	Drug and alcohol related contacts and diagnoses, police and justice related contacts for drug and alcohol issues/violence.
Mental health	Mental Health Register Emergency Department Presentations Hospital Morbidity Database Death Register	Timing and frequency of mental health contacts, diagnoses, co-morbid mental health and substance use issues, length of stay, self-harm episodes, suicide.
Pregnancy and parenting	Birth Registrations Midwives Notifications Hospital Morbidity Database Child Protection Data	Pregnancy contacts, age at birth, pregnancy complications, birth outcomes, mental health contacts, substance use contacts, child protection contacts.

### Bias reduction

Navigating Through Life is subject to a number of biases due to its complexity. Biases associated with study design will be discussed first, followed by biases that may result from analysis of qualitative data. In terms of biases associated with study design [26], there is a risk of habituation bias of quantitative outcome measures. To counter habituation bias, outcome measures have been sequenced so that measures of similar nature will not be administered next to each other. There is a particular large risk of sponsor bias given our target population of young people in OOHC [27]. We anticipate that some young people may have a negative view of the WA Department of Communities, which may skew the responses they provide. To address this bias, interviewers reiterate the independence of interviewers from the Department and maintain a neutral viewpoint throughout the interview.

The risk for social desirability bias is greater for the qualitative interviews than for data collected through quantitative outcome measures. To minimise this bias, interviewers focus on unconditional positive regard and indirect questioning, particularly for socially sensitive questions [27, 28]. Confirmation bias, one of the most pervasive forms of bias in research, will be addressed during both the interview and the data analysis phases. To minimise the natural tendencies of interviewers to make meaning and filter information based on their preconceived ideas, researchers are continually re-evaluating impressions of respondents and challenge pre-existing assumptions and hypotheses [27].

Given that young people from culturally and linguistically diverse backgrounds will comprise half of the sample, and in particular, Aboriginal young people, the risk of cultural bias is high. To minimise culture bias, researchers adopt an approach of cultural relativism by showing unconditional positive regard and being cognisant of their own cultural assumptions [29]. Furthermore, the PAG and AERG are consulted regularly on the questions and outcome measures we use to ensure cultural sensitivity and appropriateness. Furthermore, the project has employed interviewers who are of Aboriginal descent to interview Aboriginal young people in a culturally sensitive manner.

Given that this is a population-based study, there is a risk of sampling bias [30]. To address this risk, particularly the risk of omission bias, recruitment strategies are being employed so that all young people who meet the eligibility criteria are approached to participate instead of limiting recruitment to a stratified sample, as that would have made recruitment impractical. To counter this bias, the research team will monitor recruitment to ensure we recruit proportional representation of participants within three age bands (i.e., 15–17, 18–21, 22–25 years). In terms of cultural background, the proportion of Aboriginal young people in care in WA is approximately 56%, so we will adjust our recruitment strategies with that target in mind. We will also monitor the number of young people from regional and remote areas to ensure they are not under- or over-represented. Similarly, in addressing the potential for inclusion bias, we will purposefully target marginalised young people through generic youth services, such as homelessness services, to ensure that we do not only include those who are easy to reach [30]. The data linkage component of Navigating Through Life also allows us to overcome some of the issues of selection and follow-up bias that are present when researching with vulnerable populations, as all eligible young people across WA will be included in the linked data.

The risk of procedural bias is addressed by making sure participants are in no way rushed to complete the outcome measures and the interview. This is achieved by taking regular breaks and ensuring all measures are comprehensively completed and by incorporating skip logic to minimise the burden on participants [27].

Measurement bias has been limited through careful selection of the outcome measures [26]. Measures have been selected with the following considerations in mind: 1) alignment with outcomes that are of interest given the aims of the study, specifically to focus on outcomes for young people with lived experience in OOHC; 2) measures that are culturally sensitive; and 3) measures with established and robust psychometrics. The psychometric quality of all outcome measures will be analysed after data collection to ensure the measures are psychometrically robust, including checking for cross-cultural measurement invariance. Furthermore, throughout the data collection phase, researchers continuously emphasise that there are no correct or incorrect responses, and that we are only interested to understand the viewpoints of participants [30].

To address biases associated with the analysis of qualitative data, trustworthiness will be established through four strategies [29]: credibility, transferability, dependability, and confirmability. To enhance consistency, all interviews are being conducted by interviewers who have been trained in the interview approach by experienced interviewers. Throughout the data analysis, coding will be completed independently and consensus will be reached on the codes and, subsequently, on the themes. The development of sub-themes and themes will be discussed among the team of qualitative researchers until full agreement had been reached. Process interpretations will be cross-checked across several meetings involving experienced qualitative researchers who are not involved in the Longitudinal Study to add non-biased and critical independence to the analysis. A clear audit trail using thematic analysis will be maintained throughout the process. Finally, transcriptions will be sent back to a subset of approving participants for member checking, to ensure accurate recording of their responses to add further rigour to triangulation strategies.

### Data analysis

#### Quantitative data

Standard descriptive statistics (frequencies and percentages for categorical variables; means, medians, and standard deviations for variables measured on a continuous scale) will be used to summarise the profile of participants in both studies. Any differences in demographic variables between the three groups of Data Linkage Study participants (OOHC Cohort, Non-OOHC CP Cohort, non-OOHC Non-CP Cohort) will be assessed using the Chi-square, one way ANOVA or Kruskal Wallis non-parametric tests, as appropriate. Similarly, the sociodemographic variables of age, gender, cultural identity and geographic location of participants in the Longitudinal Study will be assessed against those of the OOHC Cohort to assess the representativeness of the final sample.

Drop-out from the Longitudinal Study will be examined for associations with sociodemographic variables and baseline levels in all outcome measures. The psychometric properties (i.e., internal consistency, construct validity, reliability) of all outcome measures will also be assessed across the waves. Longitudinal standardised outcome measure data from Longitudinal Study participants will be analysed using multi-level modelling. Analysis will investigate cross-lagged effects and pathways to determine stability and change in resilience, wellbeing, social inclusion, self-determination independent living skills over time. Some outcome data obtained from the Data Linkage Study will be included in the analysis.

The Data Linkage Study data will provide the information to describe the pathway that each participant takes through the primary outcomes of: mental health, education, social development, employment and independent living. With each of the outcomes classified as a binary variable (present or absent at any time during the follow-up period), a Logistic regression model will be used to identify whether the odds of each outcome differ between groups. The model will be extended to adjust for demographic variables that may be relevant, and will also identify the influence of other outcomes on each outcome of interest. For example, the model will identify the importance of a previous mental health event on the educational or employment outcomes.

The participants will be classified into groups according to their age (years) at entry to the study, so that Kaplan-Meier curves can be obtained for each age. These curves will show the time to first occurrence of each outcome for the three groups, and the Logrank test will be used to compare these curves. The results will include the median times to each outcome for the three groups, along with their 95% confidence intervals. A Cox Proportional Hazards model will be used to analyse differences between groups while taking into account demographic and baseline characteristics of each participant, as well as the occurrence of other outcome events.

Statistical analyses will be performed using the SAS [31] and MPlus software [32], and, following convention, a *p*-value < 0.05 will be taken to indicate a statistically significant association in all tests.

#### Qualitative data

The overall design for the qualitative data analysis can be described as a trajectory analysis [33]. A trajectory analysis “focuses on changes over time for an individual or small group of individuals” [33] , p. 2. This approach will allow an analysis of the qualitative data to produce themes and concepts that can be examined over different time points. Given the sample size and the longitudinal prospective design of the Longitudinal Study, two different approaches will be implemented to manage the large volume of qualitative data so that changes can be tracked at an individual and cohort level over time while also allowing for in-depth qualitative analysis.

First, individual transcripts will be coded by the interviewer using a matrix-coding tool developed by the research team. The analytical matrix tool was developed following a pilot analysis of nine transcripts (three young people in care, three who have left care, and three Aboriginal young people). The qualitative descriptors generated from the nine pilot transcripts were organised into three descriptive categories per construct area covered in the interview schedule (see Table 1 for construct areas). The three descriptive categories per construct area are based on Stein’s [34] conceptualisation of the transition experience of care leavers (i.e., struggling, surviving, moving on). The same matrix will be used to code transcripts across all waves of data collection, allowing for individual experiences to be consistently plotted from the baseline interview through to subsequent interviews, thus aiding with maintaining stability in the trajectory analysis. The analytical matrix will also be used as a tool to guide inter-coder reliability discussions among the research team throughout the data coding process, which will ensure coding consistency. Relevant contextual factors, such as specific events or factors that have influenced the experience of leaving care will be identified and thematically described at this point. Nvivo will be used for transcript archival and for specific queries and verification of data themes and patterns. The sum total of all matrix coding will enable patterns and themes within cohorts and across the longitudinal trajectory to be identified and queried. This process will generate large-scale summative thematic descriptions of the key events and influences for the whole sample.

Second, findings from the matrix coding will be combined with data from the Data Linkage Study to identify “outlier” or “extreme” cases for follow-up unstructured in-depth interviews [35]. An outlier or extreme case yields information rich data that provides deeper insights into a select aspect of the broader linked data and the themes and patterns generated from matrix-coding. This step is essentially a purposive sampling strategy focusing on a smaller number of cases from the whole sample. Using thematic data analysis, this strategy allows for examination of selected in-depth focal points, such as construct specific inquiries or specific transition experiences. It will further support theoretical analysis by connecting linked data with construct or issue specific inquiries identified from in-depth interviews of outlier cases.

### Ethical approvals

Due to the complexity of Navigating Through Life, multiple levels of ethical clearance are required. In addition, there are considerations that must be evaluated relevant to this project in relation to working appropriately with vulnerable groups (including children and young people, and Aboriginal or Torres Strait Islander people), the use of personal information (especially medical or health related data), and waiving conditions for consent.

#### Ethical approval for Longitudinal Study

The Curtin University Human Research Ethics Committee (HREC) has approved the protocol for the Longitudinal Study (HRE2018–0170). Following institutional HREC approval, approval to conduct the study with young people in the care of the WA Department of Communities was obtained. Ethical issues most pertinent to the Longitudinal Study were consent for access to linked data, consent to obtain records from Department of Communities, the risk of participant distress if sensitive topics are discussed, and the risk of participants disclosing abuse or illegal activity to researchers.

Consent for linked data and for researchers to obtain participants’ records from Department of Communities is being obtained via different methods for the two Longitudinal Study cohorts. For participants in the In-care Cohort, consent for study participation, linked data and researcher access to records was obtained by the Department of Communities CEO (their legal guardian) during the Department’s approval of the study protocol. Young people in the In-care Cohort assent to participation after being informed by researchers about the linking of their data and that their departmental records will be provided to researchers. Young people from the Exited-care Cohort are asked to provide informed written consent for linked data and access to their Department records.

To minimise the risk of causing unintended participant distress, all data collection instruments (qualitative and quantitative) were reviewed by Curtin University HREC, Department of Communities, and members of the Reference Groups and Advisory Groups. In addition, a protocol has been developed whereby researchers discuss with participants their support networks prior to commencing data collection to identify a support person to be contacted if the participant becomes distressed. In addition, researchers monitor participants for distress throughout the data collection process and cease the process should a participant become distressed.

Participant confidentiality will be maintained by researchers at all times, with the exception of disclosure of abuse, neglect, poor quality of care, or illegal activity. Wave 1 Interview questions have been designed in such a way to limit the likelihood of disclosure, and this principle will be implemented for the development of subsequent interview schedules. Prior to obtaining consent and commencing data collection researchers inform young people about instances where confidentiality cannot be maintained. A protocol for handling disclosures has been developed in conjunction with the Department of Communities.

#### Ethical approval for researcher access to linked data

Access to linked WA Government data will be achieved in conjunction with a concurrent project taking a public health approach to child abuse and neglect. Prior to that project accessing linked data, approval from multiple research governance bodies was required, namely the WA Department of Health HREC (2012/37) and University HRECs (RA/4/1/5952). In order to obtain approval of the WA Department of Health HREC approval from two other research governance bodies was first required: 1) the Research Management Group (RMG) of the Developmental Pathways Project, and 2) the Western Australian Aboriginal Health Ethics Committee (WAAHEC; approval #458). The RMG is comprised of health and non-health data custodians, all of who have agreed to the use of the data for this project. Approval from WAAHEC enables the use of Aboriginal identifiers in our analyses of linked data. The ethics committees and the departmental governance processes are in place to ensure that the protection of data is addressed and that there is justification of the benefits of the research.

## Discussion

The Navigating Through Life study offers an innovative and comprehensive methodology, suitable for researching long-term outcomes for vulnerable, marginalised and difficult to reach populations. The data generated will be rich and comprehensive, allowing for translation into evidence about the various dimensions of programs required, and how they can be targeted in order to better meet the complex and intersecting needs of our study population.

In the context of OOHC research, Navigating Through Life will result in new knowledge about young people in and exiting OOHC in Western Australia. This will be the largest study of Australian young people leaving OOHC and will increase both the depth and the breadth of our understanding of young peoples’ outcomes and related pathways into adulthood. Findings will provide policy makers, government and non-government service providers, peak industry bodies, researchers, foster carers and kinship carers, and young people with lived experience of OOHC with comprehensive knowledge and new insights not previously available.

The key intended outcome for Navigating Through Life is that the findings are translated into care leaving programs that reflect the needs of, and improve outcomes for, young people who have lived in OOHC. Involving industry partners in the research is a key dissemination and translation strategy. Most agencies in WA delivering primary services to young people transitioning from care are involved in the research, either by representation on the research team or on one of the reference and advisory groups. By having industry stakeholders so closely involved, the research team intend to disseminate findings and ideas for practice improvement directly through these established networks. In addition to influencing practice and programming, this study aims to impact policy, both at State and Federal government level. The findings of this research will also be highly relevant to other jurisdictions for developing evidence-informed programming to improve personal, societal and economic outcomes.

## Data Availability

Not applicable as article does not contain any data.

## Abbreviations

AERG: Aboriginal Expert Reference Group
CI: Chief Investigators
CP: Child protection
HREC: Human Research Ethics Committee
LEERG: Lived Experience Expert Reference Group
LSAY: Longitudinal Surveys of Australian Youth
NAT: Needs Assessment Tool
OOHC: Out-of-home care
PAG: Project Advisory Group
RMG: Research Management Group
WA: Western Australia
WAAHEC: Western Australian Aboriginal Health Ethics Committee
